# Community-Based Recreational Football: A Novel Approach to Promote Physical Activity and Quality of Life in Prostate Cancer Survivors

**DOI:** 10.3390/ijerph110605567

**Published:** 2014-05-26

**Authors:** Ditte Marie Bruun, Eik Bjerre, Peter Krustrup, Klaus Brasso, Christoffer Johansen, Mikael Rørth, Julie Midtgaard

**Affiliations:** 1 The University Hospital Centre for Health Care Research, Copenhagen University Hospital Rigshospitalet, Blegdamsvej 9, 2100 Copenhagen, Denmark; E-Mails: eik_bjerre@hotmail.com (E.B.); julie@ucsf.dk (J.M.); 2 Copenhagen Centre for Team Sport and Health, Department of Nutrition, Exercise and Sports, University of Copenhagen, Nørre Alle 51, 2200 Copenhagen, Denmark; E-Mail: pkrustrup@nexs.ku.dk; 3 Department of Sport and Health Sciences, College of Life and Environmental Sciences, University of Exeter, St Luke’s Campus, Heavitree Road, Devon, EX1 2LU, UK; 4 Copenhagen Prostate Cancer Center, Department of Urology, Copenhagen University Hospital Rigshospitalet, Blegdamsvej 9, 2100 Copenhagen, Denmark; E-Mail: klaus.brasso@regionh.dk; 5 Unit of Survivorship, Danish Cancer Society Research Centre, Strandboulevarden 49, 2100 Copenhagen, Denmark; E-Mail: christof@cancer.dk; 6 Department of Oncology, Copenhagen University Hospital Rigshospitalet, Blegdamsvej 9, 2100 Copenhagen, Denmark; E-Mail: mikael.roerth@regionh.dk; 7 Department of Public Health, University of Copenhagen, Øster Farimagsgade 5, P.O. Box 2099, 1014 Copenhagen, Denmark

**Keywords:** cancer survivors, soccer, community, exercise-based rehabilitation

## Abstract

As the number of cancer survivors continues to increase, there is an increasing focus on management of the long-term consequences of cancer including health promotion and prevention of co-morbidity. Prostate cancer is the most frequent type of cancer type in men and causes increased risk of heart disease, diabetes and osteoporosis. Epidemiological evidence points to a positive effect of regular physical activity on all-cause and prostate cancer mortality and current clinical evidence supports the use of exercise in cancer rehabilitation. However, the external validity of existing exercise studies is limited and the majority of prostate cancer survivors remain sedentary. Hence, novel approaches to evaluate and promote physical activity are warranted. This paper presents the rationale behind the delivery and evaluation of community-based recreational football offered in existing football clubs under the Danish Football Association to promote quality of life and physical activity adherence in prostate cancer survivors. The RE-AIM framework will be applied to evaluate the impact of the intervention including outcomes both at the individual and organizational level. By introducing community-based sport environments, the study offers a novel approach in the strive towards sustained physical activity adherence and accessibility in prostate cancer survivors.

## 1. Introduction

Due to the growth and aging of the population, substantial advances in the detection of cancer and improved treatments, the number of cancer survivors is growing worldwide [1]. As a consequence, there is an increasing focus on long-term health and well-being in cancer survivors, which includes prevention of late-effects, recurrence of secondary cancers, co-morbidities and chronic diseases, as well as to the organization and facilitation of behavioral interventions. It has been demonstrated that addressing modifiable health behaviors may reduce morbidities among cancer survivors [2]. In particular, regular physical activity of moderate-intensity is increasingly recognized as a strategy for health promotion and tertiary prevention in cancer survivors [3,4]. Clinical practitioners play a pivotal role in the promotion of healthy lifestyle in patients [5], yet physical activity promotion is not integrated into routine clinical practice [6]. Moreover, as there is a trend towards a general decline in the length of hospital inpatient care, a shift toward community-based prevention and health promotion is occurring [7]. In this regard, development and evaluation of optimized coordination and cooperation between hospitals and community-based organizations *i.e.* municipalities and patient organizations is warranted and currently remains a challenge in cancer survivorship care [8]. Against this background, the purpose of this paper is to present the rationale behind the delivery and evaluation of community-based recreational football as a novel approach to promote physical activity and quality of life in Danish prostate cancer survivors, *i.e.*, the FC Prostate Community Study. 

## 2. Rationale

### 2.1. Prostate Cancer

Prostate cancer is the most frequently diagnosed type of cancer in men [9]. Due to advances in detection and treatment, life expectancy in prostate cancer patients has improved and according the most recent data the relative 10 year survival rate is 99% [10]. Prostate cancer may involve prolonged (>10 years) hormone treatment *i.e.*, androgen-deprivation therapy [11] causing numerous adverse effects, including reduced bone density, decrease in muscle mass and strength, increase in body weight and fat mass and decline in physical functioning [12], all of which have an impact on quality of life and may result in, or be worsened by, sedentary behavior. Survivors of prostate cancer therefore face an elevated risk of developing of diabetes [13,14], cardiovascular diseases [15,16] and osteoporosis [17,18] and survivorship care has become a significant aspect in the provision of health care in prostate cancer survivors.

### 2.2. The Role of Physical Activity in Prostate Cancer Survivors

Although, still few in number, epidemiological studies suggest that regular physical activity of moderate intensity (>3 h/week) may have a positive effect on prostate cancer progression [19] and survival [20]. Accordingly, clinical randomized controlled trials show that exercise *i.e.*, combined resistance and aerobic exercise has a beneficial impact on treatment- and disease-related side effects [21,22]. However, comparable to the suboptimal rate of physical activity participation in healthy older adult males [23], less than half of prostate cancer survivors are meeting the official guidelines for physical activity [24]. As such, it is crucial to develop and evaluate new strategies to increase access to and maintenance of physical activity, to reduce mortality and morbidity and improve quality of life in this dramatically growing population of cancer survivors. 

### 2.3. The Potential of Sport in Male Cancer Survivorship Care

Research demonstrates that male patients have a general preference for advice rather than emotional support, and prefer that disease and health related matters take up only a small part of their daily life and identity [25]. This may explain why men, in comparison to their female counterparts, are less likely to adopt lifestyle changes and take up health messages [5], tend to conceal signs of vulnerability and weakness, and behave in concordance with traditional masculine ideals *i.e.*, demonstrations of power, control and self-reliance to protect their masculinity [26]. As an alternative to traditional psychosocial support, the facilitation of male-orientated health promotion through sport in clinical populations constitutes a promising novel research approach [27,28,29]. The implementation of lifestyle-modifying interventions outside the hospital [30] and the introduction of sport [31] may address the challenge of delivering health promotion and adoption of regular physical activity participation in male cancer survivors in a way that respect their requirements for activation oriented and rational activities [30,32]. 

### 2.4. Recreational Football as a Health Promoting Activity

Football is considered to be the most prominent team sport in the world [33] and current research is documenting the significant health promoting effects of recreational football defined as non-tournament-based small-sided games [34] in untrained men [35], men with hypertension [36] and type 2 diabetes [37]. In addition to persistent physiological effects, recreational football provides peer-based psychosocial support and social capital [38], which is likely to contribute to long-term adoption. Scandinavian countries have a strong tradition of community-based sport organizations, which provide a natural environment for team sports *i.e.*, football, and play a large role in the society as a culturally well-established and valued institution [39]. Therefore, community-based sport associations may be conducive to increasing accessibility and adherence of new groups of members and offer a unique opportunity to extend and reframe our conception of what rehabilitation should consist of and how it may be organized and implemented.

### 2.5. The Effects of Recreational Football in Prostate Cancer Patients

We recently examined the safety and efficacy of 12 weeks of recreational football in prostate cancer patients undergoing androgen deprivation treatment [40]. The results indicate that recreational football, defined as participation in tournament-based small-sided games [41], may improve lean body mass and muscle strength [42]. Moreover, a qualitative inquiry [43] documented, that the intervention was regarded by participants as a welcome opportunity to regain control and take responsibility for their own health without assuming the patient role. Hence, the recently completed FC Prostate Randomized Controlled Trial validated the relevance and efficacy of recreational football training delivered in a clinical setting for a homogenous group of prostate cancer patients. However, because of strict inclusion criteria, the external validity including the transferability potential of the intervention to a realist setting is limited. With a few exceptions [44,45], the problem of limited applicability is common in the exercise-oncology literature characterized mainly by clinical controlled trials examining the efficacy of hospital-based, time-limited and cost intensive exercise interventions. Thus, the long-term effects and adoption of exercise delivered in real-life settings remain unexplored [46].

### 2.6. The FC Prostate Community Study

To meet the growing challenge of the management of the long-term health consequences of cancer, there is an increased need for provision of novel evidence-based survivorship care strategies, which may be transferred to and implemented in real-life settings characterized by limited resources and the need to cater to large populations unable or unwilling to travel long distances. Accordingly, we aim to initiate the FC Prostate Community Study with the purpose to explore the effectiveness of community-based recreational football to promote physical activity adoption and quality of life in a heterogeneous group of prostate cancer survivors [47,48].

## 3. Framework

### 3.1. Principles

The FC Prostate Community Study is characterized by the development and delivery of a physical activity based intervention *i.e.*, recreational football training, evaluated and implemented in a real-life and permanent setting [49] *i.e.*, existing local football clubs. To support maintenance of the potential impact of the intervention beyond the study period and independent of the involvement of researchers, the study includes a time-unlimited intervention with continuous enrolment of participants, and mobilizing of local resources and partners [50] by use of existing structures for physical activity. 

### 3.2. Multi-agency Partnership

The intervention is developed and will be delivered through a multi-agency and multi-level partnership between researchers *i.e.*, behavioral and physiological scientists, clinical cancer specialists *i.e.*, The Copenhagen Prostate Cancer Centre, political stakeholders *i.e.*, The Danish Football Association [51] and The Danish Cancer Society and patient advocates *i.e.*, The Danish Prostate Cancer Organization [52]. The partners represent different agencies with independent skills, motives and objectives (Table 1), which will be brought together through a process of information sharing and recognition of mutual benefits to be gained through collaborative practice. 

**Table 1:** Partners, skills and objectives.

Agency	Partner	Skill	Objectives
Research Institutions	Copenhagen University Hospital The University of Copenhagen	Data collection and analysis	Scientific knowledge building
Clinical Departments	The Copenhagen Prostate Cancer Centre	Access to and recruitment of participants including delivery of information on safety (feed-back)	Physical activity promotion opportunity including referral to a gender-sensitive and long-term recreational physical activity program
Private and Political Stakeholders	The Danish Cancer Society The Danish Football Association	Delivery of training facilities and existing organizational structures for recreational physical activity	Attraction of new and/or underserved populations *i.e.*, male cancer survivors.
Patient Advocates	The Danish Prostate Cancer Organization	Communication with prostate cancer patients and survivors	Promotion and recognition of prostate cancer patients’ specific rehabilitation needs

### 3.3. Design

The study will use a pragmatic approach to evaluate the effectiveness of football training delivered in a real-world setting [53]. Pragmatic trials are defined as trials designed to determine the effects of an intervention under the usual conditions in which it will be applied, with the intent to inform users (*i.e.*, clinical, health service or policy agents) to choose between options for care [54]. Accordingly, the intention of pragmatic trials is to interfere as little as possible with the usual process of care delivery, which makes understanding of the social context of the intervention essential [55]. Thus, in addition to the evaluation of the effectiveness of the intervention, the FC Prostate Community Study intends to describe the distinctive features of the setting, participants, clinicians and implementation of the intervention. This is likely to enable assessment of how the context has influenced the delivery of the intervention [56] and thus enhance of the applicability of the results.

#### Research Plan

The study will be carried out in two steps: (1) a non-randomized pilot study (step I) and (2) a pragmatic randomized controlled trial (RCT) (step II). The purpose of the pilot study is to examine the feasibility of the intervention (n = 45) in three distinct clubs each including approximately 12–18 participants. On this basis, we aim to verify the relevance and validity of outcomes and instruments that will be included in the subsequent RCT. The purpose of the RCT is to evaluate the impact (*i.e.*, the reach, effectiveness, adaptation, implementation and maintenance) of the intervention. Estimation of the exact sample size needed in the RCT (step II) including choice of primary outcome will be based on pre- to post-test changes in quality of life and physical activity behavior observed in the pilot study. 

### 3.4. Intervention: Community-based Recreational Football Training

#### 3.4.1. Intervention Setting and Content

The intervention will be carried out in local football clubs and will consist of supervised recreational football training offered as one-hour session two times per week. For practical and pragmatic reasons each team will consist of 12-18 participants. To reflect normal practice in local football clubs, participants will pay a token member fee to the football clubs to be registered as club members with access to ordinary club activities during the one year study period. The intervention is intended to be applied with a high degree of inbuilt flexibility. However, it will be carried out according to a specially developed training manual containing guidance on warming up, technical drills and matches [34] to secure a slow progression of the training from moderate to high exercise intensity (average heart rates of 85%) [42]. As part of the intervention, and given evidence of the importance of regular feedback in physical activity adherence [57], participants’ physical fitness and muscle strength will be monitored every fourth week using simple field tests [58,59].

#### 3.4.2. Management of Training and Certification of Coaches

To support local implementation and utilization of knowledge and experiences drawn from this study, coaches from the football clubs will be recruited within the local football clubs to manage the training. Each coach will complete an obligatory 12-h training seminar including lectures on prostate cancer patho-physiology, review of common treatment options and side-effects, psychosocial aspects of prostate cancer, physical training of cancer patients and first aid, as well as introduction to the training manual and tests that will be used. The seminars will be led by health professionals and experienced football instructors and will draw on experiences from the clinical FC Prostate trial [40,42].

#### 3.4.3. E-communication

Participants and coaches will have access to a web-based portal *i.e.*, integrated homepage, mobile-app and data base hosting data on attendance, field testing and potential adverse events. The web-based portal constitutes a digitized two-way intervention enabling custom-fit feed-back to the participants [60] and continuous evaluation of the progression of the intervention to the coaches.

### 3.5. Target Group

The primary target group of the study is survivors of prostate cancer. With the intention of reflecting the heterogeneity of the prostate cancer survivor population and in accordance with the pragmatic attitude [54], the study will implement little selection of participants beyond the clinical indication of interest [55]. Accordingly, study participants will include prostate cancer survivors independent of stage of disease, age, place of residence and previous football experience. Participants will be recruited from the Copenhagen Prostate Cancer Center and Department of Oncology at Copenhagen University Hospital. The primary attending urologist or oncologist will present oral and written information about the study to eligible patients in accordance with the listed criteria. Patients considered unsuitable for training based on the judgment of the physician will not be included in the study. In addition to the group of prostate cancer survivors, study subjects include clinical practitioners and local football clubs as equal partners in the implementation. 

### 3.6. Evaluation

The study provides measurement of outcomes that are of immediate importance to both prostate cancer survivors and clinicians e.g., health related quality of life, physical activity maintenance. However, the study also yield outcomes that are of direct relevance to funders, political stakeholders and community agencies e.g., costs, appeal and maintenance of the target group, since these agencies are likely to be those making decisions about intervention options in the cancer rehabilitation setting in which the intervention will be implemented. 

To reflect the complexity of the intervention, the study combines quantitative and qualitative research methods and allows for investigation of both objective and subjective outcomes at various levels. Thus, the study aims to provide empirical data related both to the participants in the intervention *i.e.*, the participant level, but also to the social context (*i.e.*, setting and interventions agents), in which the intervention *i.e.*, recreational football is conducted *i.e.*, the organizational level and gives hereby a balanced emphasis to the internal and external validity of the intervention [53]. For this purpose, the study applies the RE-AIM framework [61], which is a method of multilevel evaluation of the implementation of behavioral interventions in a real-world setting [46,62,63]. RE-AIM is an acronym for Reach, Effectiveness, Adoption, Implementation and Maintenance: *Reach* refers to participation rate and representativeness of participants, *effectiveness* pertains to the impact of the intervention on specific individual outcomes e.g., quality of life and physical activity participation, *adoption* operates at the setting level and concerns the percentage and representativeness of settings that will adopt the intervention e.g., football club acceptance and readiness, *implementation* refers to intervention integrity, and the quality, consistency and costs of delivery of the intervention across settings e.g., local variations of implementation, and finally *maintenance*, which is concerned with long-term adherence of impact on individual outcomes and sustainability of the interventions in the settings [49]. The following outcomes and instruments will be included in the study:

*Reach* (participant level) of the intervention will be assessed when participants enter the study (baseline) and includes registration of:

- *Participant rates and representativeness of participants* by the means of own-developed questionnaires including data on age, marital status, socio-economic status, education, occupation and social relations and health behavior. Data on prostate cancer diagnosis, disease stage and previous treatment will be achieved from medical records, while information on general medical conditions and co-morbidity will be collected through The Danish Cancer Registry and The Danish National Patient Register.
- *Barriers for participation* by the means of from semi-structured individual interviews (n = 10–15) with men with prostate cancer, who are eligible for the study but deny participation at baseline.
- *Adherence* including reasons for non-attendance of training sessions will be recorded among intervention participants.

*Effectiveness* (participant level) of the intervention will be assessed at 3 and 6 month and include the following six outcomes:

- *Quality of life* will be measured using the questionnaire Functional Assessment of Cancer Therapy-Prostate (FACT-P) [64] covering 27 questions on physical, social, emotional and functional well-being in cancer patients in general and 12 questions specified prostate cancer patients.
- *Fatigue* will be measured using the FACIT-Fatigue Scale (FACT-F) [65] covering 13 questions on cancer anemia and fatigue [66].
- *Self-reported physical activity* will be measured using the Danish version of the Physical Activity Questionnaire (IPAQ) [67] in its short form.
- *Marital adjustment* is measured using the short form of The Dyadic Adjustment Scale (DAS-7) containing seven questions [68].
- *Physical, psychological and social well-being* will be explored using in-depth individual interviews (n = 10–15) with participants in the intervention group using purposeful selection *i.e.*, maximal variation and theoretically saturation [69]. The following topics will be covered: Group cohesion, relation to partner/spouse, intimacy.
- *Marital well-being and care giver role* will be explored using in-depth individual interviews (n = 10–15) with partners/spouses to participants in the intervention group. Interviewees are selected using maximal variation and intensity sampling [69]. The following topics will be covered: Changes in mood and behavior, marital satisfaction, intimacy, mutual exchange of care.

*Adoption* (organizational level) will be assessed when the football clubs enter the study and includes description of:

- *The number and representativeness of settings* by the means of own-developed questionnaires including data on the club profile, number of members, organization and management and geographic location.
- *Acceptance of and readiness for intervention* using focus group interviews (n = 5 × 7 − 9) and key person interviews (n = 10) with agents from the local football clubs. The purpose is to explore potential health policy or strategy, subsidies, social responsibility profile, attitudes about the intervention.
- *Preliminary conditions for referral from health professionals* using focus group interviews (n = 5 × 7 − 9) and key information interviews (n = 10) with health professionals in urological and oncological departments. The purpose is to uncover normal practice, culture and norms in health professional to gain understanding of referral to physical activity programs *i.e.* football training.

*Implementation* (organizational level) will be described after 3 and 6 months including:

- *Local variations of implementation* by the means of semi-structured individual interviews with coaches and club representatives and participant observations during football training (20 h per club during one year). The purpose is to uncover local variations and adaptations in the implementation of the intervention across the different football clubs.
- *Costs of the intervention* will be estimated from registration of direct participant (*i.e.*, travel time and costs, member fees and training equipment) and direct provider costs (*i.e.*, materials, time of information meetings and club staff meetings, time spent by the coach) [70].

*Maintenance* (participant and organizational level) will be assessed after 12-month including:

- *Long-term effectiveness* of quality of life, fatigue, self-reported physical activity and marital adjustment in participants in the intervention group.
- *Sustainability* of the intervention in the football clubs, including long-term adaption and institutionalization by the means of key informant interviews with club representatives (n = 10).

**Figure 1:**
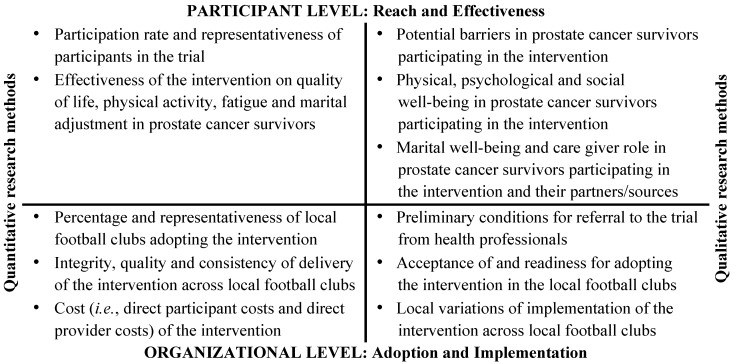
Research focus areas across the RE-AIM framework dimensions.

Figure 1 show the research focus areas across the RE-AIM framework dimensions. 

### 3.7. Data Analyses

As the objective of the current article is to describe the rationale for the FC Prostate Community study, it is not valid to report specific statistical analyses in respect to findings of the pilot study (step I), which will guide the measurements of outcomes in the RCT study (step II). To reduce bias, the study will be registered at clinicalTrials.gov and quantitative data from questionnaires, medical records and register will be hosted by Copenhagen Trial Unit (CTU). Statistical tests will be used according to data level and reported in accordance to SAMPL guidelines [71]. To reduced loss to follow-up, all participants will be contacted at endpoints and intention-to-treat analyses will be performed as primary analysis. Statistical Analysis Systems (SAS) version 9.1.4 will be used. All qualitative data will be audio-taped, transcribed and analyzed using de- and re-texualizing [72] with the intention to uncover meaning and motives for actions. Lived experiences (*i.e.*, in-depth individual interviews) will be analyzed using a descriptive phenomenological approach [73], while attitudes, behaviors and social experiences (*i.e.*, key informant interviews, focus group interviews and participant observations) will be analyzed using interpretive description [74] aimed at producing recommendations for practice.

### 3.8. Ethics and Safety

Written informed consent will be obtained prior to initiating study activities. Attendance and reasons for non-attendance of training sessions (e.g., muscle soreness and injury) will be registered for all participants in the web-based portal, accessible for coaches, participants and researchers. Serious adverse events occurring during training will immediately be reported to the Unit for Patient Safety in the Capital Region of Denmark. Minor events related to physical contact or stumbling can occur during football training, and only pain and soreness persisting for more than 24 h will be recorded.

## 4. Perspectives

The Prostate Community Study has been developed to respond to the challenges of sedentary lifestyle, limited accessibility to facilities, resources, equipment and long-term maintenance of physical activity in prostate cancer survivors. Using a pragmatic framework for evaluation of community-based football in prostate cancer survivors, the study will deliver knowledge on the impact *i.e.*, Reach, Effectiveness, Adoption, Implementation and Maintenance of the intervention. 

The key element of the study is the introduction of community-based sport environments as potential contexts and strategies for increasing physical activity in male cancer survivors [31]. Participation in sport is increasingly recognized as important for public health, and the physiological and psychological benefits of sport participation within the general population have been widely accepted [75], supporting the potential of sport to address some of the health challenges within clinical populations. Moreover, the literature demonstrates an emerging focus on men’s health [76] and the facilitation of health education and promotion in male-specific contexts and settings e.g., sport clubs and stadiums [27,28,29]. In the light of research indicating that men prefer to reduce their focus on disease and health care provision in hospitalized contexts [25], the introduction of innovative settings dissociated from hospitals and institutionalized health care centers may provide a strategy for male-orientated health promotion, with the potential of attracting men, who are traditionally difficult to access via health promotion activities [77]. Recently, Hunt *et al.* 2014 showed the effectiveness of a weight loss and health living program delivered through Scottish Premier League football clubs in overweight and obese men, which highlighted the unique potential of a gender sensitized context, content and style of delivery [29].

To our knowledge this will be the first study to evaluate the impact of recreational sport *i.e.*, football in the context of cancer survivorship care. Recent studies have described the use of football in the context of rehabilitation of groups unfamiliar with football [43,78]. Hence, a study by Mynard *et al.* [78] demonstrated how participation in a community-based football team can provide therapeutic benefits, such as sense of inclusion, achievement and enjoyment, in socially and occupationally disadvantaged people and indicates that occupational therapy can be effectively conducted outside the health-care center and may complement existing welfare-based approaches [78]. 

These findings support recent research on non-clinical exercise programs for cancer survivors delivered in community settings [79]. A small number of studies based on partnerships between local communities, hospitals and research units indicate that community-based exercise programs are feasible, may positively affect disease and treatment related outcomes [80] and may overcome well-known barriers for participation in health promotion in cancer survivors, such as travel distance and costs [81].

The intervention will be delivered through a multi-agency partnership in a real-world setting *i.e.*, local football clubs with the intention of supporting persistence of the intervention beyond the study period. Thus, the study complies with the trend towards community-based and multidisciplinary delivery of cancer survivorship care [7]. 

In Scandinavian countries the municipalities are responsible for organising cancer rehabilitation at a general level in Denmark [8]. In addition, Scandinavian countries have a strong tradition for community-based patient and sport organizations, which play an important role in society, as culturally well-established and highly valued institutions [39]. Sport organizations in Scandinavian countries are easily accessible in the communities where people live and are characterized by a high level of participation compared to most other countries. Moreover, a large proportion of sporting activities are organized by voluntary sport associations with a high level of interaction and interdependence with public authorities at national, regional and local levels [82]. For these reasons, community-based sport environments may offer a unique opportunity to contribute to reframing and extending existing concepts of hospital-based rehabilitation, which are often time-limited and involve a specific number of activities for each patient. 

It is well known that men and therefore constitute an underserved population in cancer rehabilitation [83]. Specifically, the FC Prostate Community Study has the potential to reshape existing approaches to respond to the challenge of the limited number of male cancer patients in traditional rehabilitation (15% men *vs*. 85% women) [84]. Thus, although some types of competitive contact sports (e.g., rugby or American football) may not be appropriate given the skills and physical abilities required, as well as the possibility for injury [31], the potential advantages of sport in the context of male cancer rehabilitation are worth capitalizing on.

As stated previously, the safety and efficacy of recreational football in prostate cancer patients have been established previously under strict and highly controlled conditions in a hospital environment in a randomized clinical controlled trial (the FC Prostate RCT) including 57 selected patients undergoing androgen deprivation treatment. Unlike this recently conducted study, the purpose of the present study is to evaluate the feasibility and effectiveness of recreational football when delivered in a real-life setting characterized by dynamic processes and limited resources. The study is characterized by a complex intervention [85] requiring evaluation across different levels *i.e.*, prostate cancer survivors, local football clubs, and clinical departments and various mechanisms *i.e.*, physiological and psychosocial [86], requiring different methodological considerations. We suggest that only by evaluating the intervention in the same setting likely to ultimately implement the intervention, *i.e.*, local football clubs, we will be able to support the external validity of the intervention including the evaluation of the transferability, applicability and dissemination potential. The use of simple but valid field tests, the fact that the study participants are expected to pay a member fee to the club similar to regular club members, and the fact that coaches are expected to be recruited within the existing club staff, means that the proposed intervention may be administrated with minimal provision human or financial resources, which is regarded as fundamental for the sustainability of the intervention beyond the study period [87]. Moreover, the proposed and ongoing monitoring of the local adaption and implementation of the intervention by the means of the RE-AIM framework including assessment of fidelity to the training manuals and registrations of costs is likely to further support the external validity of the study. 

The number of cancer survivors will increase in the coming years, largely because of the aging population and ongoing improvements in cancer care [88]. Utilization of innovative settings and partnerships in cancer survivorship care will be essential for sustained provision and thus long-term health and disease-free survival. The FC Prostate Community Study will contribute to this process by offering novel knowledge about a unique, male-orientated health promotion strategy *i.e.*, football delivered in a real life setting *i.e.*, community-based football clubs. 

## 5. Conclusions

Community-based recreational football constitutes a complex intervention involving numerous methodological considerations. However, although the evaluation and implementation poses great challenges, the potential for positive impact on quality of life and physical activity adherence in a growing population of cancer survivors indicates the relevance of this novel, highly generalizable and applicable approach in cancer rehabilitation. By mobilizing existing resources in local communities, this study supports future implementation and dissemination of sport interventions e.g., football in survivorship care.
